# Genetics of the Pig Tapeworm in Madagascar Reveal a History of Human Dispersal and Colonization

**DOI:** 10.1371/journal.pone.0109002

**Published:** 2014-10-15

**Authors:** Tetsuya Yanagida, Jean-François Carod, Yasuhito Sako, Minoru Nakao, Eric P. Hoberg, Akira Ito

**Affiliations:** 1 Department of Parasitology, Asahikawa Medical University, Asahikawa, Hokkaido, Japan; 2 Institut Pasteur de Madagascar, Antananarivo, Madagascar; 3 US Department of Agriculture, Agricultural Research Service, US National Parasite Collection, Animal Parasitic Diseases Laboratory, Beltsville, Maryland, United States of America; Kunming Institute of Zoology, Chinese Academy of Sciences, China

## Abstract

An intricate history of human dispersal and geographic colonization has strongly affected the distribution of human pathogens. The pig tapeworm *Taenia solium* occurs throughout the world as the causative agent of cysticercosis, one of the most serious neglected tropical diseases. Discrete genetic lineages of *T. solium* in Asia and Africa/Latin America are geographically disjunct; only in Madagascar are they sympatric. Linguistic, archaeological and genetic evidence has indicated that the people in Madagascar have mixed ancestry from Island Southeast Asia and East Africa. Hence, anthropogenic introduction of the tapeworm from Southeast Asia and Africa had been postulated. This study shows that the major mitochondrial haplotype of *T. solium* in Madagascar is closely related to those from the Indian Subcontinent. Parasitological evidence presented here, and human genetics previously reported, support the hypothesis of an Indian influence on Malagasy culture coinciding with periods of early human migration onto the island. We also found evidence of nuclear-mitochondrial discordance in single tapeworms, indicating unexpected cross-fertilization between the two lineages of *T. solium*. Analyses of genetic and geographic populations of *T. solium* in Madagascar will shed light on apparently rapid evolution of this organism driven by recent (<2,000 yr) human migrations, following tens of thousands of years of geographic isolation.

## Introduction

The pig tapeworm *Taenia solium* (Cestoda: Taeniidae) is an etiologic agent of cysticercosis, an important zoonosis and neglected tropical disease, and recently ranked as the most important food-borne parasites on a global scale [1]. The lifecycle of *T. solium* includes humans as the only definitive hosts and domestic pigs as principal intermediate hosts. Cysticercosis refers to infection of various tissues of swine or humans with cysticerci larvae due to ingestion of eggs released from people harboring adult worms in the intestine. Cysticercosis of the central nervous system (neurocysticercosis or NCC), warrants special attention because it is a major cause of seizures and epilepsy in endemic areas [2] and can be lethal especially in remote areas of developing countries [3]. *T. solium* is distributed worldwide where local people consume pork without meat inspection. We previously reported that *T. solium* can be divided into two mitochondrial (mtDNA) genetic linages, Asian and Afro-American which differ in the clinical manifestations of human cysticercosis [4]. Their distributions are geographically disjunct in Asia or Africa and Latin America [4]. It has been postulated that *T. solium* emerged from Africa with early modern humans and through geographic expansion became distributed initially across Eurasia prior to the advent of agriculture and domestication of swine [5]–[7]. Phylogenetic studies have suggested that divergence of the two lineages occurred in the Pleistocene [4], [6], [8]. Recently, sympatry of both mitochondrial lineages was confirmed in Madagascar [6], [8].

Madagascar is a country known to be hyper-endemic for cysticercosis [9], [10]. Cysticercosis in pigs results in condemnation of carcasses, particularly in heavy infections, and thus constitutes a considerable economic challenge. Understanding the current distribution for these parasites and the historical factors involved in geographic colonization of Madagascar can contribute insights of importance in developing a capacity for control and mitigation of infections in swine and human hosts.

Malagasy people are divided into 18 ethnic groups and have diverse cultures. Surprisingly, the first human settlement occurred approximately 2000 years ago as one endpoint of Austronesian migration. Linguistic and archeological evidence suggests that the Malagasy people have mixed ancestry from Island Southeast Asia (ISEA), especially Borneo, and from East Africa [11]; dual origins confirmed by analyses of mtDNA and nuclear DNA [12]. In addition, a contribution to the gene pool of Malagasy people from India has recently been suggested by mtDNA genetic analysis [13]. Prehistoric human migrations can also be traced by parasitological evidence. For example, archaeoparasitology of some intestinal parasites have indicated the existence of human migration routes into the New World other than those involving Bering Land Bridge [14]. Phylogenetic analysis suggested that *T. solium* has been introduced into Madagascar multiple times from a number of different areas [8], but the dynamics of these introductions and establishment were not fully elucidated. In the present study, reciprocal insights for the distributional history of hosts and parasites emerge from an exploration of *T. solium* and human occupation of Madagascar.

Historically disjunct populations of *T. solium* are now in sympatry in Madagascar, affording a unique opportunity to explore the possibility of cross-fertilization and hybridization as a fundamental process among cestodes, and concurrently reflect on the degree of isolation and distinct nature of these genotypes. Cestodes are hermaphrodites with two potential modes of reproduction, self- and cross-fertilization. *T. solium* has often been referred to as a self-fertilizer because it is nearly always found alone in the human intestine. However, random amplified polymorphic DNA showed heterozygosity in cysticerci of *T. solium*, suggesting cross-fertilization between different individual worms [15]. Consequently, it may be assumed that the two genotypes of *T. solium* can cross-fertilize in infections involving multiple adults, which may occur early in the infection process. Analysis of maternal inherited mtDNA alone, however, is not sufficient to examine putative hybridization events. Thus, we initially established nuclear DNA markers to differentiate geographic variation in *T. solium*. Secondarily, genetic polymorphism of *T. solium* in Madagascar was investigated to clarify whether hybridization occurs on the island.

## Materials and Methods

### Parasite isolates and DNA sequencing

During 2005 to 2008, 57 pigs slaughtered from 16 different localities in 5 provinces on Madagascar were found positive for *T. solium* cysticerci. No specific permissions were required for the field survey, and it did not involve endangered or protected species. Meat inspectors in each province were requested to collect infected pig meats at slaughterhouses from the various locations. Pigs were regularly slaughtered at the official slaughterhouses of each city (Table S1), and the slaughtering was controlled by meat inspectors according to the regulations of the Republic of Madagascar. Pigs were sacrificed for routine slaughterhouse purposes and not for research purposes. When positive for *Taenia* cysticerci, infected meats were cut and inserted into sterile containers, and sent to the Pasteur Institute of Madagascar within 24 hours. Then the cysticerci were extracted and washed at the laboratory, and frozen at −20°C until use. All samples were then fixed with 70% ethanol and shipped to Japan according to the research agreement between Pasteur Institute of Madagascar and Asahikawa Medical University. One or two cysts from each pig were subjected to molecular analysis. The genomic DNA of each cyst was extracted by DNeasy blood and tissue kit (Qiagen), and subsequently used as a template for polymerase chain reaction (PCR). For the mtDNA gene markers, the entire cytochrome *c* oxidase subunit I (*cox1*) and cytochrome *b* (*cob*) were amplified by PCR using previously reported primer pairs [4]. PCR products were treated with illustra ExoStar (GE Healthcare) to remove excess primers and dNTPs, and directly sequenced with a BigDye Terminator v3.1 and a 3500 DNA sequencer (Life Technologies).

Nuclear gene markers including RNA polymerase II second largest subunit (*rpb2*), phosphoenolpyruvate carboxykinase (*pepck*), DNA polymerase delta (*pold*) and a low-molecular-weight glycoprotein antigen (*Ag2*) were amplified using primer pairs published previously [16], [17]. These nuclear genes were chosen because they have been shown to be useful for the molecular phylogeny of taeniid tapeworms including species of *Taenia* (*rpb2, pepck* and *pold*) or for differentiating geographic genotypes of *T. solium* (*Ag2*). Initially, 41 geographic isolates of *T. solium* from 14 countries were used to investigate the geographical variability of nuclear gene markers. PCR products were sequenced with the same protocols as mtDNA gene markers. When geographical variations were found, new primers were designed to amplify the short fragments including mutation sites in order to reduce the cost and labor. PCR was performed in 20 µL volumes containing 0.5 units of Ex Taq Hot Start Version (TaKaRa, Japan), 0.2 mM of dNTP, 1×Ex Taq Buffer with a final MgCl_2_ concentration of 2.0 mM, 15 pmol of each primer and 1.0 µL of genomic DNA. PCR amplification consisted of initial denaturation of 94°C for 2 min, 35 cycles of 94°C for 15 sec, 55°C for 15 sec and 72°C for 30 sec, and a terminal extension at 72°C for 1 min. In cases of double peaks in the sequencing of nuclear genes, PCR products were ligated into pGEM-T plasmid vector (Promega) and then introduced into *Escherichia coli* DH5α. At least 10 clonal colonies were picked from an agar plate and their insert DNAs were sequenced to confirm allelic polymorphism.

### Data analysis

Nucleotide sequences of the mitochondrial *cob* (1068 sites) and *cox1* (1620 sites) were concatenated into a total sequence (2688 sites). They were aligned by Clustal W 2.0 [18] with those sequences available in public databases. Amino acid sequences were inferred with reference to the echinoderm mitochondrial genetic code [19]. Pairwise divergence values among the obtained nucleotide sequences were calculated using the MEGA5 package [20] using Kimura's two parameter model with a γ-shaped parameter (α = 0.5). The identification of mtDNA haplotypes and the drawing of their network was computed by TCS 1.2 software [21] using statistical parsimony [22]. Evaluation of the rate of outcrossing was based on an estimate of the inbreeding coefficient for each nuclear locus and deviation from Hardy-Weinberg proportions as *F* = 1-*Hobs*/*Hexp*, where *H* is the actual population heterozygosity and *Hexp* is the expected heterozygosity under H – W equilibrium.

## Results

### Mitochondrial DNA phylogeography

In the present study, we collected 109 cysticerci larvae from 57 pigs across 5 provinces on Madagascar. In total, 8 haplotypes (MDG1 to MDG8) of concatenated *cox1* and *cob* genes were detected. When compared with individual genes, the numbers of haplotypes were reduced to 3 (*cob*) and 7 (*cox1*). All the nucleotide sequences of each haplotype are deposited in GenBank with accession numbers AB781355-AB781364. The frequency of the nucleotide substitution was 1.6% (17 sites/1068 sites) in *cob* and 1.4% (22/1620) in *cox1* (Tables S2 and S3). Among 39 point mutation sites identified, 24 (61.5%) were synonymous and 15 (38.5%) were non-synonymous substitutions. The maximum value of divergence among the 8 haplotypes was 1.4%. Among the mtDNA gene sequences of *T. solium* deposited in the public databases, 14 sets of the complete *cob* and *cox1* gene sequences were concatenated and used for the haplotype network analysis together with those from Madagascar (Table 1). These sequences were chosen because they had unequivocal published references allowing confirmation that the sequences of the two genes were obtained from one individual parasite.

**Table 1:** Mitochondrial haplotypes of *T. solium* used for the phylogeographic analysis.

Haplotypes^a^	Localities	Accession numbers		References
		*Cox1*	*Cob*	
MDG1	Madagascar	AB781355	AB781362	This study
MDG2	Madagascar	AB781356	Same as MDG1	This study
MDG3	Madagascar	Same as MDG1	AB781363	This study
MDG4	Madagascar	AB781357	Same as MDG1	This study
MDG5	Madagascar	AB781358	Same as MDG1	This study
MDG6	Madagascar	AB781359	Same as MDG1	This study
MDG7	Madagascar	AB781360	AB781364	This study
MDG8	Madagascar	AB781361	Same as MDG7	This study
CHN1	China	AB066485	AB066570	Nakao *et al*. 2002^4^
CHN2	China	AB066486	AB066571	Nakao *et al*. 2002^4^
ID-BA	Bali, Indonesia	AB631045	Not determined	Swastika *et al*. 2012^24^
ID-PA	Papua, Indonesia	AB066488	AB066573	Nakao *et al*. 2002^4^
IND	India	AB066489	AB066574	Nakao *et al*. 2002^4^
NPL1	Nepal	AB491985	AB781746	Yanagida *et al*. 2010^23^
NPL2	Nepal	AB491986	Same as MDG1	Yanagida *et al*. 2010^23^
THA	Thailand	AB066487	AB066572	Nakao *et al*. 2002^4^
BRA	Brazil	AB066492	AB066577	Nakao *et al*. 2002^4^
CMR	Cameroon	Same as MEX1	AB066579	Nakao *et al*. 2002^4^
ECU	Ecuador	AB066491	AB066576	Nakao *et al*. 2002^4^
MEX1	Mexico	AB066490	AB066575	Nakao *et al*. 2002^4^
MEX2	Mexico	FN995657	FN995661	Michelet & Dauga 2012^6^
MEX3	Mexico	FH995658	FN995662	Michelet & Dauga 2012^6^
TZA	Tanzania	AB066493	AB066578	Nakao *et al*. 2002^4^

a The mitochondrial haplotypes were determined based on the concatenated nucleotide sequences of complete *cox1* (1620 bp) and *cob* (1068 bp), except for ID-BA.

**Figure 1:**
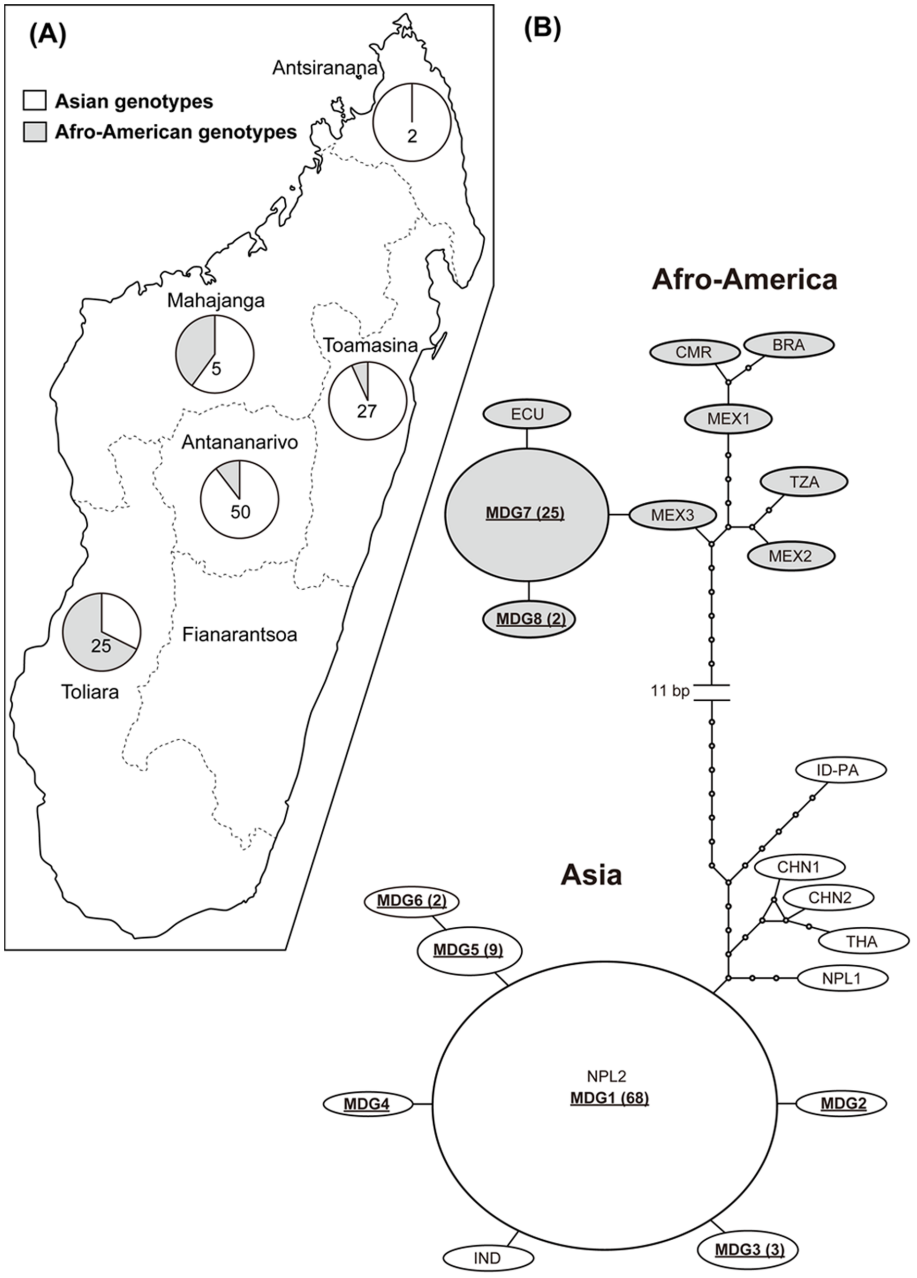
Mitochondrial genotypes of *T. solium* in Madagascar. (A) Pie charts illustrating the frequencies of the Asian and Afro-American mitochondrial genotypes of *T. solium* in each collection site. The numbers in the charts show the sample size for parasite isolates examined. Madagascar is divided into the 7 former provinces. (B) The haplotype network of concatenated mtDNA gene sequences. The size of the ellipses is roughly proportional to the haplotype frequency, and the actual numbers of haplotypes (>1) are enclosed in parentheses.

Network analysis clearly showed these 8 haplotypes are divided into two genotypes (Fig. 1). Six haplotypes (MDG1-6) were the Asian genotype and the remaining two (MDG7-8) were the Afro-American genotype. Overall, 77% (84/109) of the Madagascan haplotypes were the Asian genotype (Table 2). The Asian genotype was found in all examined provinces and was generally dominant except in Toliara. The Afro-American genotype was identified in 4 of 5 examined localities. Among the haplotypes obtained, MDG1 was the major (62%), followed by MDG7 (23%). Among 52 pigs in which two cysts were examined, the different haplotypes were simultaneously obtained in 3 hosts; Asian and Afro-American haplotypes (MDG1 and MDG7) were identified from two hosts, and the different Asian haplotypes (MDG1 and MDG4) were obtained from one host. MDG1 was 100% identical to the haplotype obtained from a pig in Nepal [23], and one base different from the Indian haplotype. All Asian haplotypes from Madagascar are grouped with those from the Indian Subcontinent. On the other hand, these Asian haplotypes were distantly related to that from Papua, Indonesia. Further, the *cox1* haplotype of the isolate from Bali Island, Indonesia [23] was also distantly related. In contrast, MDG7 was one base different from MDG8 and the haplotypes from Mexico, Ecuador, Bolivia.

**Table 2:** Genotypes of *T. solium* in Madagascar at mitochondrial DNA and each nuclear locus.

Provinces	No. of pigs	No. of cysts	No. of mtDNA haplotypes								No. of genotypes at each nuclear locus								
			Asian						Afro-American		*Ag2*			*rpb2*			*pold*		
			MDG1	MDG2	MDG3	MDG4	MDG5	MDG6	MDG7	MDG8	*A/A*	*A/B*	*B/B*	*A/A*	*A/B*	*B/B*	*A/A*	*A/C*	*C/C*
Antananarivo	26	50	44	0	0	1	0	0	5	0	46	1	3	49	1	0	47	2	1
Toamasina	14	27	13	0	1	0	9	2	2	0	27	0	0	27	0	0	27	0	0
Mahajanga	3	5	3	0	0	0	0	0	2	0	5	0	0	4	1	0	4	1	0
Toliara	13	25	8	1	0	0	0	0	14	2	6	3	16	6	2	17	7	5	13
Antsiranana	1	2	0	0	2	0	0	0	0	0	2	0	0	2	0	0	2	0	0
Total	57	109	68	1	3	1	9	2	23	2	86	4	19	88	4	17	87	8	14

### Nuclear DNA

Among the *Ag2*, *rpb2* and *pold* locus, two (*Ag2* and *rpb2*) or three (*pold*) alleles were confirmed from the 14 geographical isolates from 14 countries; no geographical variation was found in the *pepck* locus. Subsequently, *Ag2*, *rpb2* and *pold* were chosen as appropriate nuclear DNA markers to discriminate the Asian and Afro-American genotypes of *T. solium*. To amplify the target regions including the variable sites, new primers were designed for *rpb2* and *pold* (Table S4). An additional 27 geographical isolates were analyzed using these new primer sets, to confirm geographical variation. *Ag2A*, *rpb2A* and *poldA* (Asian alleles) were only found in Asia and *Ag2*B, *rpb2B*, *poldB* and *poldC* (Afro-American Alleles) were obtained from Latin American and African countries (Table 3). The sequence difference among the alleles was 1–3 bp. All the nucleotide sequences of each allele of *rpb2* and *pold* are deposited in GenBank with accession numbers AB781365-AB781369.

**Table 3:** Distribution of alleles at each nuclear locus around the world.

Localities	No. isolates examined	Alleles		
		*Ag2*	*rpb2*	*pold*
China	4	*Ag2A*	*rpb2A*	*poldA*
Thailand	2	*Ag2A*	*rpb2A*	*poldA*
Papua, Indonesia	2	*Ag2A*	*rpb2A*	*poldA*
Nepal	3	*Ag2A*	*rpb2A*	*poldA*
India	4	*Ag2A*	*rpb2A*	*poldA*
Vietnam	1	*Ag2A*	*rpb2A*	*poldA*
**Asian total**	16			
Tanzania	7	*Ag2B*	*rpb2B*	*poldB*
Mozambique	7	*Ag2B*	*rpb2B*	*poldB*
South Africa	2	*Ag2B*	*rpb2B*	*poldC*
Cameroon	4	*Ag2B*	*rpb2B*	*poldC*
Mexico	1	*Ag2B*	*rpb2B*	*poldB*
Ecuador	2	*Ag2B*	*rpb2B*	*poldC*
Peru	1	*Ag2B*	*rpb2B*	*poldC*
Brazil	1	*Ag2B*	*rpb2B*	*poldC*
** Afro-American total**	25			

Establishment of nuclear DNA markers allowed us to investigate possible hybridization events in Madagascar. All three nuclear genes were amplified and sequenced for the same 109 cysts as mtDNA genes. Overall, the Asian alleles were the majority in Madagascar with frequencies of 0.81–0.84 (Table 2). Asian alleles were the majority in all the examined regions except for Toliara, and the frequencies of Afro-American alleles in the region were 0.60–0.72. No new alleles were identified among these three loci. Among 12 cysts, the nucleotide sequences of one or more loci could not be determined by direct sequencing because of double peaks in the sequence electropherograms. As the result of cloning of the polymorphic PCR amplicons, two alleles were detected at an approximate ratio of 1∶1. These cases were considered to be heterozygous in each locus. Two cysts obtained from one pig were heterozygous at the all three loci examined. Twenty-two cysts possessed discordant mitochondrial and nuclear genotypes, Asian and Afro-American, in at least one nuclear locus (Table 4). The inbreeding coefficient (*F*) was estimated only for the sub-population in Toliara because of the considerably biased allele frequency in the other sub-populations; at this locality, *F* was equal to 0.86 (*Ag2*), 0.90 (*rpb2*) and 0.79 (*pold*).

**Table 4:** Genotypes of *T. solium* showing nuclear-mitochondrial discordance.

ID of samples	MtDNA haplotype^a^	Genotype at each locus^a^^,^ b			Localities
		*Ag2*	*rpb2*	*pold*	
TsolMDG21b	MDG1	***B*** **/** ***B***	***B*** **/** ***B***	*A*/*A*	Toliara
TsolMDG29a	MDG1	***B*** **/** ***B***	*A*/*A*	*A*/*A*	Toamasina
TsolMDG62a	MDG1	*A*/*A*	*A*/*A*	***C*** **/** ***C***	Antananarivo
TsolMDG62b	MDG1	*A*/*A*	*A*/*A*	*A* /***C***	Antananarivo
TsolMDG67a	MDG1	***B*** **/** ***B***	***B*** **/** ***B***	*A* /***C***	Toliara
TsolMDG68b	MDG1	***B*** **/** ***B***	***B*** **/** ***B***	***C*** **/** ***C***	Toliara
TsolMDG04a	**MDG7**	***B*** **/** ***B***	*A* /***B***	*A*/*A*	Antananarivo
TsolMDG04b	**MDG7**	***B*** **/** ***B***	*A*/*A*	*A*/*A*	Antananarivo
TsolMDG12b	**MDG7**	*A*/*A*	*A*/*A*	*A* /***C***	Antananarivo
TsolMDG13a	**MDG7**	*A* /***B***	*A*/*A*	*A*/*A*	Antananarivo
TsolMDG13b	**MDG7**	***B*** **/** ***B***	*A*/*A*	*A*/*A*	Antananarivo
TsolMDG25a	**MDG7**	*A* /***B***	*A* /***B***	*A* /***C***	Toliara
TsolMDG25b	**MDG7**	*A* /***B***	*A* /***B***	*A* /***C***	Toliara
TsolMDG28a	**MDG7**	*A*/*A*	***B*** **/** ***B***	***C*** **/** ***C***	Toliara
TsolMDG37a	**MDG7**	*A*/*A*	*A*/*A*	*A*/*A*	Toamasina
TsolMDG37b	**MDG7**	*A*/*A*	*A*/*A*	*A*/*A*	Toamasina
TsolMDG50a	**MDG7**	*A*/*A*	*A* /***B***	*A*/*A*	Mahajanga
TsolMDG50b	**MDG7**	*A*/*A*	*A*/*A*	*A* /***C***	Mahajanga
TsolMDG21a	**MDG7**	***B*** **/** ***B***	***B*** **/** ***B***	*A* /***C***	Toliara
TsolMDG68a	**MDG7**	***B*** **/** ***B***	***B*** **/** ***B***	*A* /***C***	Toliara
TsolMDG69a	**MDG7**	*A* /***B***	***B*** **/** ***B***	***C*** **/** ***C***	Toliara
TsolMDG69b	**MDG7**	***B*** **/** ***B***	***B*** **/** ***B***	*A* /***C***	Toliara

a Haplotypes and alleles in bold are Afro-American ones.

b Genotypes with underline indicate those at heterozygous loci.

## Discussion

The sympatric distribution of Asian and Afro-American mitochondrial genotypes was confirmed on Madagascar, corroborating a prior report [6], [8]. Although the Afro-American mitochondrial genotype previously was identified only in Toliara [8], we confirmed the co-occurrence of Asian and Afro-American genotypes in 4 out of 7 provinces, indicating a widespread distribution for the two mitochondrial genotypes across the island. Major genotypes differed geographically and across provinces. The Asian genotype was generally dominant at all localities except in Toliara, where 64% of the parasite isolates were the Afro-American genotype.

Differences in the distribution of the dominant genotypes of *T. solium* among provinces can be attributed to disparate history and ethnic origins in each region and patterns of human dispersal and migration over the past several thousand years. Phylogenetic analyses of *Taenia* have suggested a relatively deep origin in Africa for *T. solium*, which may have initially parasitized hominin ancestors of modern humans in the early Pleistocene following a host-switching event from large carnivores [5], [7], [25]. It has been postulated that *T. solium* emerged from Africa with early modern humans and through geographic expansion became distributed initially across Eurasia prior to the domestication of swine which now represent a primary intermediate host [5], [6]. Although there is no direct evidence, phylogenetic studies using mtDNA markers have suggested the divergence of the two genotypes, now associated respectively with Africa/America and with southern Asia/Indian Subcontinent occurred in the Pleistocene [4], [6], [8].

The dominant haplotype in Madagascar (MDG1) demonstrates Asian affinities and is genetically most similar to those from Nepal and India, but distantly related to that from Papua, Indonesia. Further, a *cox1* gene sequence of the isolate from Bali Island [24] was distantly related to MDG1 and other haplotypes from Madagascar. Consequently, it appears that the origin of the Asian genotype on Madagascar is not from ISEA, coincidental with the first human immigrants, but from the Indian Subcontinent. Although Asian origins of the Malagasy people have generally been linked to immigrants and populations from ISEA, our result and recent report on human mitochondrial genetics [13] indicate the importance of Indian influence on the diversity of people and culture in Madagascar consistent with and reflecting a history of human dispersal within the past 2,000 years.

On the other hand, the dominant Afro-American haplotype in Madagascar (MDG7) is closely related to those from Mexico and Ecuador. It does not imply a direct link for Madagascan and Latin American populations, because it is apparent that Afro-American haplotypes have been widely disseminated and the same haplotype can be obtained from both African and Latin American countries [4], [8]. It was suggested that *T. solium* was introduced into Latin America from Europe or Africa coincidental with European expansion and development of maritime trade routes after the 15th century [4], [26]. The dominance of the Afro-American genotype at Toliara, where the current populace is primarily of African descent, suggests that parasites were introduced to Madagascar, probably recurrently, with people and swine from coastal East Africa in a time frame within the past hundreds of years, although clarification requires further study of isolated populations in areas bordering the Mozambique Channel.

Both Asian and Afro-American genotypes on Madagascar showed a simple network with the major (MDG1 and MDG7) and satellite haplotypes. This result indicates a minimum of two independent events of anthropogenic introduction for *T. solium* from historically disjunct geographic regions in relatively shallow ecological time. It is not clear whether *T. solium* was introduced with infected pigs or humans, but it is reasonable to consider that establishment occurred after the first human settlement 2000 years ago because humans are the only definitive hosts. Phylogeography of swine has revealed the distribution of different haplogroups among South Asia, mainland Southeast Asia and ISEA, resulting from Neolithic, human-mediated translocation [27], [28]. Thus, genetic analysis of the pigs in Madagascar may shed light on how the tapeworm dispersed across the Indian Ocean.

In the present study, nuclear-mitochondrial discordance was confirmed in all three loci examined, suggesting hybridization between individual worms possessing different genotypes in the recent past. Two cysts from a pig in Toliara were heterozygous at all three loci, suggesting these were F1 hybrids between Asian and Afro-American populations; this genotype could appear at the F2 or later generation by self-fertilization of a hybrid-derived individual worm. Nuclear-mitochondrial discordance in *T. solium* has been confirmed only in Madagascar to date, indicating the hybridization event occurred on the island. The inbreeding coefficient *F* of the sub-population in Toliara was about 0.8–0.9. If *F* is interpreted as the rate of selfing [29], it means that 10–20% of the parasite individuals in the subpopulation are outcrossing. The frequency of outcrossing is much less than that demonstrated in another taeniid tapeworm *Echinococcus granulosus*, which were estimated as 74% [30]. Such a contrast is consistent with extraordinarily large infrapopulations typical of *E. granulosus* in canid definitive hosts and thus the chance of mating is simply higher than that of *T. solium*. Nevertheless, the estimated rate of outcrossing for *T. solium* was unexpectedly high when considering that these tapeworms are nearly always found in single-worm infections in humans. However, we experienced a case of taeniasis involving 20 *T. solium* adults in China [31], and we assume that the multiple infection of *T. solium* tapeworms is not so rare in endemic areas. Our result suggests that the chance of outcrossing has been underestimated and establishes hybridization as a common outcome for the Asian and Afro-American genotypes in zones of contact or sympatry. Further epidemiological study on taeniasis in Madagascar may contribute to a better understanding of the breeding systems of *T. solium*.

## Conclusions

In the present study, we show that *T. solium* was introduced and established on Madagascar at least twice in the past 2000 years. An Asian origin, from the Indian Subcontinent, for some genotypes of *T. solium* contrasts with the established history and ancestry of the Malagasy culture primarily from ISEA. Our results demonstrate that tapeworms from geographically disjunct regions in Africa or Latin America and the Indian Subcontinent are now in secondary contact on Madagascar following a history of isolation for populations that may extend to the Pleistocene. Parasites with origins in Africa/Latin America or Asia reflect the complex history of development of the Malagasy culture, and in this case provide compelling evidence for the history of human occupation of the island. Our study highlights the importance of elucidating the determinants for distributions of human pathogens and is especially relevant given manifestation of distinct disease syndromes and socioeconomic impact associated with the two recognized genotypes of *T. solium* [4], [32].

## Supporting Information

Table S1 **Location of the slaughterhouses and the numbers of pigs and cysts examined in each location.**
(DOC)

Table S2 **Nucleotide substitutions of mitochondrial** ***cob*** **gene in 22 haplotypes of** ***T. solium*** **.**
(DOC)

Table S3 **Nucleotide substitutions of mitochondrial** ***cox1*** **gene in 23 haplotypes of** ***T. solium*** **.**
(DOC)

Table S4 **PCR primer pairs used for the amplification of nuclear gene markers.**
(DOC)
