# Supplementary material for: Genetics of the Pig Tapeworm in Madagascar Reveal a History of Human Dispersal and Colonization
Source: PLoS One. 2014 Oct 15;9(10):e109002. doi: 10.1371/journal.pone.0109002 (PMC4198324; doi:10.1371/journal.pone.0109002)
Supplement: Table S1 — Location of the slaughterhouses and the numbers of pigs and cysts examined in each location. (DOC) [file pone.0109002.s001.doc]

**Table S1. Location of the slaughterhouses and the numbers of pigs and cysts examined in each location.**

| Provinces | Cities | No. of pigs | No. of cysts |
| --- | --- | --- | --- |
| Antananarivo | Antananarivo | 19 | 37 |
|  | Arivonimamo | 3 | 6 |
|  | Analavory | 1 | 2 |
|  | Antanifotsy | 1 | 2 |
|  | Ambohitanibe | 1 | 2 |
|  | Morarano | 1 | 1 |
|  |  |  |  |
| Toamasina | Mahanoro | 9 | 17 |
|  | Ampasambazimba | 2 | 4 |
|  | Bejofo | 2 | 4 |
|  | Anbatondrazaka | 1 | 2 |
|  |  |  |  |
| Mahajanga | Namakia | 1 | 1 |
|  | Mahajanga | 1 | 2 |
|  | Mandritsara | 1 | 2 |
|  |  |  |  |
| Toliara | Toliara | 10 | 19 |
|  | Avaradrova | 3 | 6 |
|  |  |  |  |
| Antsiranana | Tanambao | 1 | 2 |
