# Supplementary material for: Genetics of the Pig Tapeworm in Madagascar Reveal a History of Human Dispersal and Colonization
Source: PLoS One. 2014 Oct 15;9(10):e109002. doi: 10.1371/journal.pone.0109002 (PMC4198324; doi:10.1371/journal.pone.0109002)
Supplement: Table S2 — Nucleotide substitutions of mitochondrial cob gene in 22 haplotypes of T. solium . (DOC) [file pone.0109002.s002.doc]

Table S2. **Nucleotide substitutions of mitochondrial *cob* gene in 22 haplotypes of *T. solium*.**
