# Supplementary material for: Genetics of the Pig Tapeworm in Madagascar Reveal a History of Human Dispersal and Colonization
Source: PLoS One. 2014 Oct 15;9(10):e109002. doi: 10.1371/journal.pone.0109002 (PMC4198324; doi:10.1371/journal.pone.0109002)
Supplement: Table S3 — Nucleotide substitutions of mitochondrial cox1 gene in 23 haplotypes of T. solium . (DOC) [file pone.0109002.s003.doc]

Table S3. **Nucleotide substitutions of mitochondrial *cox1* gene in 23 haplotypes of *T. solium*.**
