# Supplementary material for: Genetics of the Pig Tapeworm in Madagascar Reveal a History of Human Dispersal and Colonization
Source: PLoS One. 2014 Oct 15;9(10):e109002. doi: 10.1371/journal.pone.0109002 (PMC4198324; doi:10.1371/journal.pone.0109002)
Supplement: Table S4 — PCR primer pairs used for the amplification of nuclear gene markers. (DOC) [file pone.0109002.s004.doc]

Table S4. **PCR primer pairs used for the amplification of nuclear gene markers.**

| Target genes | Primers (5’-3’) | Amplicon size | References |
| --- | --- | --- | --- |
| Low molecular weight antigen 2 (*Ag2*) | F: CTCGCTCTCAGTGTTTTCGT | 362 bp | Sato *et al*. 2011^16^  Sato *et al*. 2011^16^ |
|  | R: TTGACAAGTTAAGCAGCTTC |  |  |
|  |  |  |  |
| RNA polymerase II second largest subunit (*rpb2*) | F: TGTCGCATCTTCGACGGTTGAAT | 504 bp | This study  This study |
|  | R: GAGCATAAGCTTGCCCTTCTCGA |  |  |
|  |  |  |  |
| DNA polymerase delta (*pold*) | F: CCTCTGCTCCTGTTGGATAAA | 560 bp | Knap *et al*. 2011^15^  This study |
|  | R: CATGGTAAGAGTCCCTTGCGAAT |  |  |
